# Giant cell myocarditis in an 18-year-old patient with new-onset systemic lupus erythematosus: A fatal case of rapidly progressing heart failure

**DOI:** 10.1177/09612033261465053

**Published:** 2026-07-02

**Authors:** Lisa C. Harling, Max Wacker, Arjang Ruhparwar, Alexander Schmeisser, Jens Wippermann

**Affiliations:** 1 Department of Cardiothoracic Surgery, 39067 University Hospital Magdeburg, Otto-von-Guericke-University, Magdeburg, Germany; 2 Aortic Institute, Yale University School of Medicine, New Haven, CT, USA; 3 Department of Cardiothoracic Surgery, Transplantation and Vascular Surgery, 9177 Hannover Medical School, Hannover, Germany; 4 Department of Internal Medicine, Division of Cardiology and Angiology, 39067 University Hospital Magdeburg, Otto-von-Guericke-University, Magdeburg, Germany

**Keywords:** cardiovascular disease, systemic lupus erythematosus, giant cell myocarditis

## Abstract

**Background:** Giant cell myocarditis (GCM) is a rare, and often fatal subtype of myocarditis, with limited reports of association with systemic lupus erythematosus (SLE). **Purpose:** We are first to report a unique case of an 18-year-old patient presenting with biventricular heart failure and newly-onset SLE, ultimately diagnosed with GCM. **Analysis/Results:** The patient had a 6-month prodrome of fatigue, fever, and malaise, followed by acute decompensation. Echocardiography showed a severely reduced left ventricular ejection fraction (5%). Serology confirmed SLE, endomyocardial biopsy identified multinucleated giant cells, confirming GCM. Despite immediate initiation of extracorporeal membrane oxygenation (ECMO) and aggressive immunosuppressive therapy (methylprednisolone, antithymocyte globulin, cyclosporine), cardiac function did not recover. The patient received a biventricular assist device (BiVAD) as intermediate-term bridge-to-transplant. However, significant complications occurred in the form of multiorgan failure, thromboembolism, and hemodynamic deterioration, ultimately leading to death before transplantation was possible. **Conclusions:** This case underscores the importance of differentiating lupus myocarditis from GCM, given the dramatically different prognosis and treatment strategies. Early differential diagnosis via biopsy is essential, particularly in young patients with rapidly progressing cardiac decline and concurrent autoimmune serologies. Aggressive immunosuppression remains the cornerstone of treatment, attempting to reduce myocardial damage and the resulting need for transplantation. Clinicians should maintain suspicion of GCM in lupus patients with cardiac compromise to initiate timely interventions.

## Key Learning Points

○ Giant cell myocarditis (GCM) is a rare but fatal differential diagnosis of lupus myocarditis.
○ Gold standard for diagnosis of GCM is histopathologic evaluation of biopsy specimens, followed by rapid immunosuppressive therapy.
○ Biventricular heart failure with poor response to steroids in lupus patients should prompt suspicion for GCM.
○ In young women with nonspecific symptoms, clinicians should actively suspect autoimmune diseases and perform a thorough examination, including electrocardiography, at an early stage.

## Introduction

Most cases of myocarditis are self-resolving and expected to make a full recovery, yet some types of myocarditis can lead to life-threatening cardiogenic shock.^1^ Giant cell myocarditis (GCM) is a rare and fatal subtype of myocarditis that can be associated with autoimmune disorders and is predominantly found in young to middle-aged adults.^1^ Treatment of GCM requires an aggressive regime consisting of multiple immunosuppressive drugs, however, this may not be sufficient for a complete recovery. The combination of immunosuppressive medication with mechanical circulatory support often reveals to be crucial for survival.^2,3^

Myocarditis can occur as a manifestation of systemic lupus erythematosus (SLE) but can be challenging to identify, as it frequently remains subclinical (discovered in 57% of postmortem autopsies), while only being clinically detected in around 9% of patients.^4^ Out of the most common autoimmune rheumatologic conditions associated with cardiovascular disorders (SLE, rheumatois arthritis, Takayasu’s arteritis, eosinophilic granulomatosis with polyangiitis, giant cell arteritis, polymyositis, dermatomyositis, antiphospholipid syndrome), SLE has the highest rate of cardiac complications (31%).^5^

We report a case of an 18-year-old patient presenting with biventricular heart failure, newly-onset SLE, and concomitant GCM. Few reports of GCM in lupus patients are found in the medical literature and we report the first case of GCM in newly-onset SLE in an extremely young patient.

Both SLE myocarditis and GCM may present with similar primary symptoms, yet recognition is crucial to initiate appropriate treatment.

## Case report

**Figure 1.**
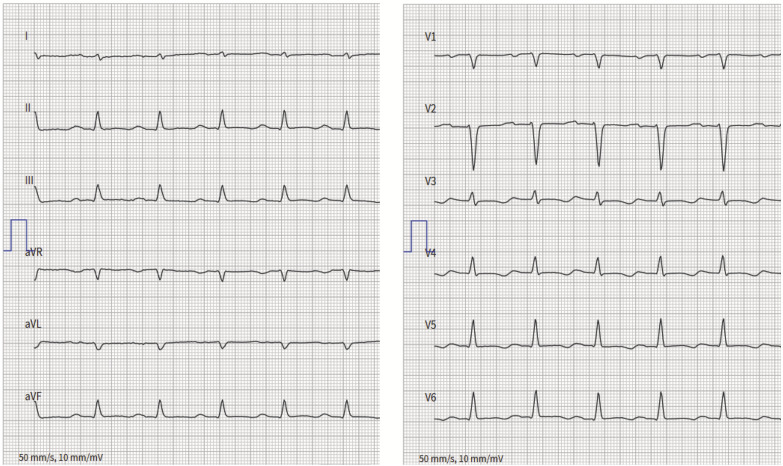
Electrocardiography from admission of the patient at first presentation. EKG paper speed 50 mm/s. Sinus rhythm, heart rate 149 bpm, normal cardiac axis, PR interval duration 0.14 s, QRS duration 0.075 s, normal R wave progression (transition point V2 to V3), no ST elevation, T wave inversion in V3 to V6.

An 18-year-old female patient was transferred to our clinic after developing cardiogenic shock. The patient had initially presented with fatigue and fever to a local hospital. She had been experiencing malaise for a total of 6 months prior, as well as exertional dyspnea and rapid weight loss for the last 3 months. Her previous medical history only included a prescription for iron supplements due to anemia. An electrocardiogram (EKG) performed at admission only showed minimal cardiac involvement (Figure 1).

**Figure 2.**
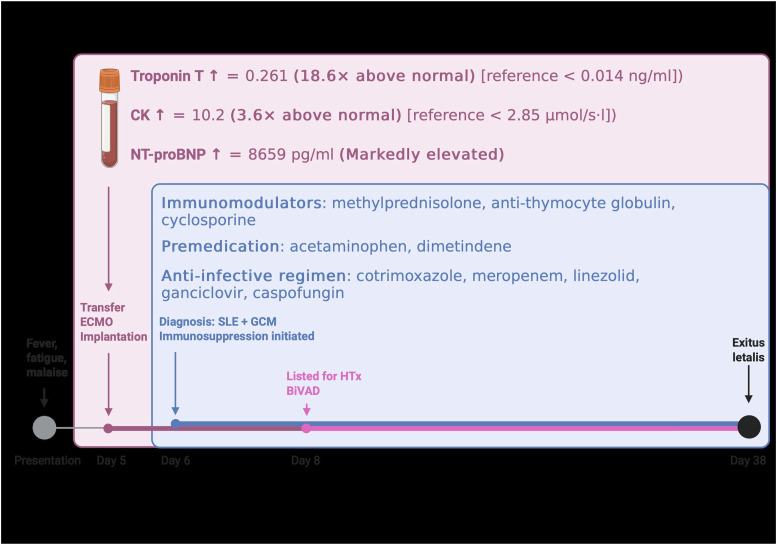
Clinical course summary. CK: creatine-kinase, ECMO: extracorporeal membrane oxygenation, NT-proBNP: N-terminal pro brain natriuretic peptide. Created in BioRender. Wacker, M. (2026) https://BioRender.com/mrlqy9y.

Before transfer, 750 mL of pericardial effusion had been drained. Echocardiography showed an LVEF (left-ventricular ejection fraction) of 5%. Laboratory values at presentation showed elevated troponin T (0.261 [<0.014 ng/ml]), and creatine-kinase (CK) (10.2 [<2.85 µmol/s·l]), as well as elevated CK-MB (no quantitative value due to hemolysis), suggesting myocardial necrosis, as well as elevated N-terminal pro brain natriuretic peptide (NT-proBNP) (8659 pg/ml), pointing towards heart failure (Figure 2). Due to the severity of her condition, the decision for ECMO (extracorporeal membrane oxygenation) and diagnostic myocardial biopsy was made by the heart team. Intraoperatively, the patient required high doses of catecholamines (noradrenaline 1.4 μ/kg/min). A pericardial effusion (200 mL), pleural effusion (600 mL), and ascites (1000 mL) were relieved once more. Antegrade ECMO implantation was performed and a myocardial biopsy was taken. Postoperatively, the patient was transferred to the intensive care unit (noradrenaline 0.8 μ/kg/min; intubated; ECMO running on 4,8 L/min) and remained hemodynamically unstable with a LVEF of 5–10%. Surgical revision was necessary to control for severe bleeding, evident in the increased output of the mediastinal drainage, as well as recurrent pericardial effusion. Packing provided primary care until secondary closure of the thorax was possible 2 days postoperatively.

**Figure 3.**
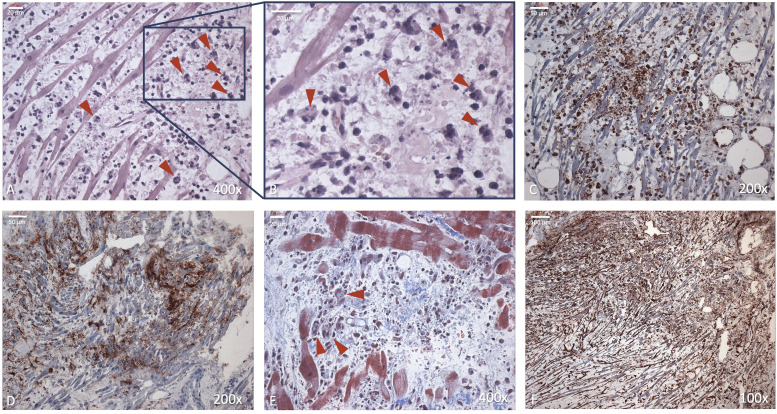
Immunohistopathologic evaluation of the myocardial biopsy. (a): Kryo hematoxylin-eosin stains of myocardial biopsy [400x], (b): Magnification of a section of (a), marked by frame, (c): CD45RO antibody [200x], (d): CD11b/Mac-1 antibody [200x], (e): Azan stain [400x], (f): HLA-DR antibody [100x]. Infiltrates of lymphocytes, plasma cells, histiocytes and eosinophiles with scattered multinucleated giant cells (exemplary marked with red arrowheads), associated with myocyte necrosis and degeneration, visible through distortion and irregular outlines of myocytes. Multinucleated giant cells in (e) (red arrowheads) showing nuclei arranged in ‘horseshow’ formation, typical for Langhans-type multinucleated giant cells. (Images reproduced with permission from the Institute for Cardiac Diagnostic and Therapy in Berlin).

A preliminary diagnosis of SLE was made on the basis of notably elevated autoantibodies (anti-nuclear antibody (ANA) presenting a titer of 1:640 [<1:80] and a homogeneous pattern; anti-double-strand (ds)DNA-IgG 19.9 [>20 IU/mL]). The 2019 EULAR/ACR was calculated as 15 (fever, thrombocytopenia, pericardial and pleural effusion, low C3 and C4 (Table 1), anti-dsDNA-IgG was not included in this calculation despite the marginal value), fulfilling the criteria for SLE.^6^ A secondary diagnosis of GCM was later confirmed in a myocardial biopsy. Liver and kidney function declined (elevated creatinine of 119 [45 – 84 µmol/l] and urea of 19.4 [2.9 – 7.5 mmol/l], as well as reduced estimated glomerular filtration rate (eGFR) (CKD-EPI) of 57 [≥ 90 ml/min/1.73 m^2^]; reduced albumin of 26.6 [35.0 – 52.0 g/l], and markedly elevated liver enzymes: alanine-aminotransferase (ALAT) 18.2 [0.17 - 0.58 µmol/s·l], aspartate-aminotransferase (ASAT) 70.1 [0.17 – 0.60] [µmol/s·l], gamma-glutamyl-transferase (GGT) 1.87 [0.10 – 0.70] [µmol/s·l], glutamate dehydrogenase (GLDH) 12,805 [<80 nmol/s·l]), resulting in thrombocytopenia (67 [176 – 391 Gpt/l]) and elevated hepatobiliary enzymes, with additional signs of pancreatitis (lipase elevated to 31 times the norm) (Table 1). The patient presented no apparent skin findings congruent with the diagnosis of SLE. Biopsy specimens were sent out for immunohistopathologic evaluation, showing infiltrates of CD11b+/Mac-1+ macrophages, CD45RO+ T memory cells and were also positively stained against HLA-DR antibodies, all three associated with GCM^2^ (Figure 3(c)–(f)). Multinucleated giant cells, characteristic of GCM, were observed in hematoxylin-eosin- and azan-stained samples (Figure 3(a), (b), (d)).

**Table 1.** Laboratory values.

​	Day 5	Day 5	Day 5	Day 5	Day 6	Day 7	Day 8
	12 AM	5 AM	12 PM	6 PM	5 AM	5 AM	5 AM
**Complete blood count**							
WBC [3.9 - 10.4 Gpt/l]	23.4 +	/	17.3 +	10.3	8.04	9.21	10.8 +
Neutrophiles [40 – 75%]	78.2 +	/	/	/	/	/	/
Lymphocytes [17 – 47%]	11.1 −	/	/	/	/	/	/
Monocytes [4.0 – 12%]	4.50	/	/	/	/	/	/
Platelets [176 – 391 Gpt/l]	102 −	/	67 −	107 −	60 −	54 −	73 −
**Cardiac enzymes**							
Troponin T hs [<0.014 ng/mL]	0.261 +	/	/	/	/	/	/
CK [<2.85 µmol/s*L]	10.2 +	/	7.39 +	5.36 +	6.81+	5.99 +	4.30 +
CK-MB [<0.40 µmol/s*L]	H	/	1.01 +	0.510 +	0.480 +	0.410 +	0.340
CK-MB/CK [<6 %]	H	/	13.7 +	9.51 +	7.05 +	6.84 +	7.91 +
Myoglobin [<58.0 ug/L]	435 +	/	/	/	/	/	/
NT-proBNP [pg/mL]	8659 +	/	/	5541 +	5457 +	/	/
**Biochemistry**							
ALAT [0.17 – 0.58] [µmol/s·l]	18.2 +	/	11 +	4.8 +	4.41 +	3.76 +	2.74 +
ASAT [0.17 – 0.60] [µmol/s·l]	70.1 +	/	/	/	/	/	/
ALP [0.58 – 1.74] [µmol/s·l]	1.59	/	1.17	/	/	/	/
γ-GTP [0.10 – 0.70] [µmol/s·l]	1.87 +	/	0.94 +	0.62	0.68	0.95 +	0.97 +
GLDH [<80] [nmol/s·l]	12805 +	/	/	/	/	/	/
LDH [2.25 – 3.55 µmol/s·l]	41.1 +	/	36.2 +	18.3 +	18.9 +	>	14.0 +
Bilirubin [<21] [µmol/l]	20.9	/	24.6 +	39.2 +	55.2 +	73.8 +	49.7 +
Bilirubin (direct) [<5] [µmol/l]	/	/	/	/	/	/	47 +
Lipase [<1.00 [µmol/s·l]	31.22 +	/	18.03 +	13.30 +	9.86 +	4.93 +	3.09 +
Creatinine [45 – 84] [µmol/l]	119 +	/	105 +	95 +	69	67	56
eGFR (CKD-EPI) [≥ 90] [ml/min/1.73 m^2^]	57 −	/	66.6 −	75 −	110	114	131
Albumin [35.0 – 52.0] [g/l]	26.6 −	/	17.9 −	33.8 −	32 −	21.8 −	24.3 −
Urea [2.9 – 7.5] [mmol/l]	19.4 +	/	16.9 +	16.3 +	12.9 +	13.4 +	13.1 +
**Coagulation tests**							
INR [<1.15]	1.33 +	/	1.38 +	1.11	1.13	1.2	1.17
aPTT [<34.4] [sec]	33.6 +	/	94.3 +	55.3 +	34.2	37.5 +	54 +
Thrombin time [<18.5] [sec]	/	/	>180 +	/	/	71.7 +	>180 +
Fibrinogen [1.50 – 4.00] [g/l]	/	/	1.11 −	2.46	3.37	3.7	3.68
Antithrombin [>80] [%]	/	/	47 −	83	78 −	73 -	80
D-dimer [<0.5] [mg/l (FEU)]	/	/	9.03 +	3.58 +	3.78 +	3.88 +	6.65 +
**Serology**							
CRP [<5] [mg/l]	35.55 +	/	63.33 +	58.81 +	94.62 +	95.39 +	45.18 +
PCT [<0.5] [ng/ml]	1.39 +	/	1.56 +	/	1.98 +	/	0.8 +
IL-6 [<7.0] [pg/ml]	310 +	/	/	/	142 +	/	/
C3 [0.9 – 1.8] [g/l]	/	0.147 −	/	/	/	/	0.357 −
C4 [0.1 – 0.4] [g/l]	/	0.047 −	/	/	/	/	0.107
IgG [5.95 – 13.10] [g/l]	/	12.5	/	/	/	/	/
ANA-IgG [<1:80] [titer]	/	1:640 +	/	/	/	/	/
Anti-dsDNA-IgG [<20] [IU/ml]	/	19.9	/	/	/	/	/
CCP-Antibody [<10 U/ml]	/	12.5 +	/	/	/	/	/
PR3-ANCA [<10 U/ml]	/	2.5	/	/	/	/	/
MPO-ANCA [<5 U/ml]	/	0.9	/	/	/	/	/
**Arterial blood gas**							
pH [7.37 – 7.45]	7.42	7.39	7.42	7.47 +	7.43	7.35 −	7.31 −
pCO_2_ [4.30 – 6.10 kPa]	3.43 −	3.48 −	4.57	4.21 −	4.48	4.95	5.20
pO_2_ [9.50 – 13.9 kPa]	10.6	14.4 +	18.8 +	31.3 +	18.7 +	14.8 +	17.7 +
O_2_ saturation [95 – 98.5 %]	94.8 −	98.2	98.9 +	99.8 +	99.1 +	98.3	98.9 +
Bicarbonate [21 – 26 mmol/l]	16.3 −	15.3 −	21.5	22.9	22.0	19.9 −	18.9 −
Standard-bicarbonate [21.0 – 26.0 mmol/l]	19.1 −	17.9 −	22.6	24.5	23.3	20.6 −	19.2 −
BE [mmol/l]	−7.300	−8.700	−2.300	−0.300	−1.700	−4.700	−6.300
Anion gap [8 – 16 mmol/l]	4.50 −	12.9	9.00	10.5	7.10 −	2.50 −	0.400 −
Lactate [<2.2 mmol/l]	3.0 +	5.1 +	4.8 +	2.8 +	1.4	1.3	1.0
Hb [7.40 – 10.0 mmol/l]	7.40	6.80 −	4.80 −	4.80 −	6.00 −	6.80 −	7.30 −

Relevant laboratory values. Norm-values in brackets. Elevated levels of NT-proBNP (>300 pg/mL) support diagnosis of acute heart failure. ANA: anti-nuclear antibodies, ANCA: antineutrophil cytoplasmic antibody (PR3: proteinase3, MPO: myeloperoxidase), anti-dsDNA: anti-double-strand deoxyribonucleic acid, ALAT: alanine-aminotransferase, ASAT: aspartate-aminotransferase, ALP: alkaline phosphatase, aPTT: activated partial thromboplastin time, BE: base excess, C: complement, CCP: cyclic citrullinated peptide, CK: creatinine-kinase, CRP: C-reactive protein, ESR: erythrocyte sedimentation rate, γ-GTP: γ-glutamyl trans-peptidase, GLDH: glutamate dehydrogenase, H: hemolysis (causing unreliable result), Hb: hemoglobulin, Ig: immunoglobulin, INR: international normalized ratio, IL: interleukin, LDH: lactate dehydrogenase, PCT: procalcitonin, WBC: white blood cells.

**Figure 4.**
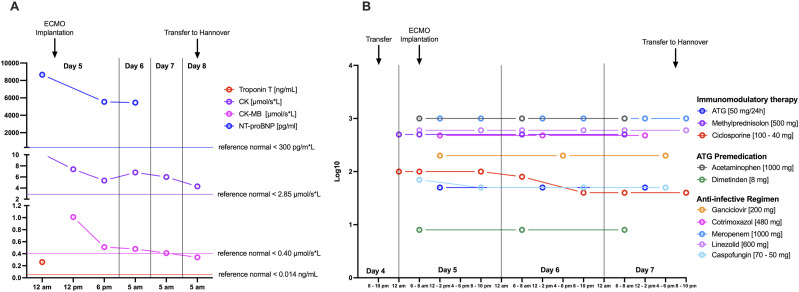
(a): Graph representing the development of cardiac enzymes over the clinical course. Lines showing normal reference values. (b): Graph representing immunosuppressive therapy regimen given over the clinical course. Methylprednisolone was started immediately after transfer of the patient to our institution (day 0). ATG (anti-thymocyte globulin) and cyclosporine were added as systemic immunomodulators treating GCM the following day (day 1). Premedication for the administration of ATG was done using acetaminophen (1000 mg) and dimetindene (8 mg). An additional anti-infective regimen (cotrimoxazole, meropenem, linezolid, ganciclovir, caspofungin) prevents infections as adverse effects of the immunosuppressive therapy.

Induction of immunomodulatory therapy (methylprednisolone) was started after the diagnosis of SLE was confirmed. ATG (anti-thymocyte globulin) and cyclosporine were added as systemic immunomodulators treating GCM. Premedication for the administration of ATG was done using acetaminophen and dimetindene. An additional anti-infective regimen (cotrimoxazole, meropenem, linezolid, ganciclovir, caspofungin) was initiated to prevent infections as adverse effects of the immunosuppressive therapy (Figures 2 and 4).

Despite timely initiation of the immunomodulatory therapy, cardiac function did not stabilize (LVEF remained 5–10%; CK, CK-MB, and NT-proBNP elevated (Table 1, Figure 4). The patient was transferred to the closest transplant center (Hanover medical school) to be listed for a heart transplant (HTx) and received a biventricular assist device (BiVAD) as a bridge-to-transplant (Excor adult, Berlin Heart GmbH, Berlin, Germany), because multiorgan failure (MOF) had advanced to a point, where ECMO as short-term bridging was not sufficient anymore. Even though myocardial function improved with the treatment, prominent MOF complicated the recovery process. Despite sufficient anticoagulation (continuous perfusion with heparin), an ischemic stroke (peripheral M1 occlusion) had to be treated by interventional thrombectomy, and thereafter, cranial hemorrhage occurred. No indication of device-associated embolization was identified. Heparin therapy was discontinued. Anticoagulation was reinitiated after 3 days (continuous argatroban infusions). Heparin induced thrombocytopenia type II and antiphospholipid syndrome were excluded as diagnoses.

Ultimately, it was not possible to stabilize the patient amid MOF, embolic complications and hemodynamic deterioration and she passed in the presence of her family before being able to receive a HTx, mere weeks after her first presentation.

## Discussion

A distinct relationship between the development of GCM and SLE is not established and has only been reported in single case reports.^7^ The only previously presented case occurred in a patient with a preexisting diagnosis of SLE (10 years prior), while we describe the first known case of a concomitant diagnosis of GCM and SLE.^7^ SLE has been observed to have the highest rate of cardiac complications in autoimmune rheumatologic conditions, specifically when examining cardiogenic shock (31%).^5^

SLE myocarditis and GCM have a similar presentation, with a good primary response to immunosuppressive therapy, but a vastly different prognosis.^5,7^ In a study published in 1997, based on international case reports of 63 patients with GCM, the rate of death or HTx was 89% and the median survival after onset of symptoms was 5.5 months.^1^ This represents a significantly worse prognosis compared with lymphocytic, such as SLE-associated, myocarditis, which has a mortality rate of 19%, or presumed viral myocarditis.^1,8,9^ Advancements in treatment and diagnosis have decreased the rate of death or HTx with GCM to 58% in recent years, yet the prognosis remains unfavorable.^8^

The initial presentation of GCM frequently begins with non-specific, flu-like symptoms and then rapidly progresses to a severe clinical presentation with congestive heart failure (75%), ventricular arrhythmias (14%), or 2^nd^ to 3^rd^ degree atrioventricular block (5%).^1^ Markers reflecting myocardial injury (increased Troponin T, necrosis, or fibrosis in myocardial biopsy) or cardiac disfunction (reduction in LVEF, or increased NT-proBNP) are associated with worse outcomes.^8^ The unspecific preclinical symptoms seen in our patient (myalgia, exertional dyspnea, fever, weight loss, anemia) may be both attributable to the undiagnosed SLE, or represent first signs of GCM. The initial EKG showed minimal cardiac involvement, seen as tachycardia and T wave inversion (Figure 1). Laboratory evaluation showed elevated Troponin T and NT-proBNP, LVEF was dramatically reduced, and necrosis was visible in biopsy specimens, all pointing towards the unfavorable clinical course of our patient.

Immunosuppressive therapy remains the cornerstone of current GCM treatment. In the presented case, the patient was initially treated with methylprednisolone alone, targeting the newly diagnosed SLE. However, as this was not sufficient in preventing further deterioration of the patient, an additional aggressive regimen of ATG and cyclosporine was added, after GCM was diagnosed in EMB. Treatment with certain combinations of immunosuppressant agents has been shown to be superior to treatment without immunosuppression or single corticosteroid therapy in the regression of heart failure.^1,3,10,11^ Myocardial inflammation appears mediated by T cells and giant cells. Therapy directed at T cells may therefore be of benefit, as suggested by reports of treatment with cyclosporine and muromonab-CD3.^9,10^ Among 22 patients treated with immunosuppressive medications that included cyclosporine, the average transplant-free survival was 13 months compared with only 3 months among 30 patients who did not receive treatment.^1^ These results were confirmed in a prospective uncontrolled study of 11 patients with GCM who were treated with 1 year of cyclosporine and glucocorticoids; nine patients additionally received 7 to 10 days of muromonab-CD3.^11^ Among 11 patients, there was only one death and two transplantations during 1-year follow-up. A benefit from immunosuppressive therapy was also suggested by a study of 32 patients with GCM, including 26 patients treated with combined immunosuppression (two to four drugs; including cyclosporine in 20 patients).^10^ Current research is looking into targeted therapy using monoclonal antibodies, such as muromonab, for specific antigens, supplementing cyclosporin in the treatment of GCM but further research will be needed to determine the efficacy of this treatment approach.^12^ Although immunosuppressive therapy can prolong survival, HTx remains the only definitive treatment option and discontinuation of immunomodulatory therapy can lead to recurrence of GCM.^1,3^

Ventricular assist device implantations are performed regularly and often the combination of mechanical support and medical therapy is necessary to prolong survival until transplantation is possible.^3,13^ However, a full recovery and weaning of mechanical support is extremely rare.^13^ Antegrade ECMO, which was utilized in our case, needs surgical placement via sternotomy and is more invasive than other modes of ECMO installation, nevertheless, it has some distinct advantages. Antegrade arterial return ensures favorable perfusion flow to the heart and brain, as well as adequate left ventricle decompression, in addition to allowing simultaneous EMB.^14^ In cases, where sternotomy is not performed simultaneously, a minimally invasive approach utilizing veno-arterial ECMO can be considered, potentially including a percutaneous left ventricular assist device, such as Impella, to facilitate left ventricle unloading.^15^ Following admission to Hanover Medical School, the initially utilized antegrade ECMO system was replaced with a Berlin Heart Excor (BiVAD) system, directly bypassing the ventricles to provide cardiac output support, without oxygenating or removing carbon dioxide from the blood.^14^ Given the patient’s initial presentation of MOF and the necessity for sufficient time for recovery, while the patient was listed for HTx, an intermediate-term bridge-to-transplant strategy was initiated using the Excor system. This approach has been shown to have superior survival rates until transplantation than the short-term bridge-to-transplant ECMO strategy, as evidenced by a study in 2009.^16^

The reduction in cardiac output (LVEF 5–10%) resulted in a decline in systemic perfusion in our patient, which in turn led to a compromise in organ function. As young patients are often able to compensate for low cardiac output and symptoms are frequently masked for an extended period, the diagnosis of declining cardiac function is often delayed. The unspecific preclinical symptoms that our patient experienced, especially exertional dyspnea, could be interpreted as first signs of a reduced cardiac output. The reduced cardiac output subsequently led to MOF and pancreatitis, and while these symptoms were initially stabilized, they ultimately complicated her recovery. Our patient additionally experienced a cerebrovascular accident, one of the main adverse device-associated events.^12^ The Excor system leads to fewer cerebrovascular events, with 50% occurring after the patients were switched to Excor after ECMO treatment.^16^ Patients with SLE have been shown to have an increased procoagulant profile due to basal activation of platelets.^17^ Similarly, myocarditis has been shown to increase coagulation via the release of proinflammatory mediators and necrosis of cardiac cells, potentially leading to the formation of mural thrombi.^18^ This is represented in the coagulation markers seen in the presented patient before the commencement of immunomodulatory therapy (INR 1.38 [<1.15], aPTT 94.3 [<34.4 s], thrombin time >180 [<18.5 s], fibrinogen 1.11 [1.50 – 4.00 g/l], antithrombin 47 [>80%], D-dimer 9.03 [<0.5 mg/l (FEU)]) (Table 1). The higher risk of thrombotic events in patients with SLE and myocarditis results in the need for prophylactic anticoagulation. Our patient was initially administered an anticoagulation regimen consisting of heparin perfusion, which was subsequently replaced with argatroban following the occurrence of thrombotic events, accompanied by hemorrhaging. Despite heparin-induced thrombocytopenia and antiphospholipid syndrome being ruled out in our patient, the anticoagulation-regimen was modified based on clinical indications, such as a decreased thrombocyte count.

We present the rare case of GCM in a young patient with newly-diagnosed SLE that resulted in biventricular heart failure. Our case suggests that in young women with nonspecific symptoms, clinicians should actively suspect autoimmune diseases and perform a thorough examination, including electrocardiography, at an early stage. Aggressive immunosuppressive treatment might prevent further deterioration and the need for HTx, yet the prognosis of GCM remains poor.

## Final diagnosis

Giant cell myocarditis in a patient with newly-diagnosed systemic lupus erythematosus.

## Data Availability

All data underlying this article is available in the article.*

## Appendix

### Abbreviations

ATG: anti-thymocyte globulin
BiVAD: biventricular assist device
CK: creatin-kinase
ECMO: extracorporeal membrane oxygenation
EKG: electrocardiogram
EMB: endomyocardial biopsy
GCM: giant cell myocarditis
HTx: heart transplantation
LVEF: left-ventricular ejection fraction
MOF: multiorgan failure
NT-proBNP: N-terminal pro brain natriuretic peptide
SLE: systemic lupus erythematosus
